# Understanding managers’ and scientists’ perspectives on opportunities to achieve more evolutionarily enlightened management in conservation

**DOI:** 10.1111/eva.12631

**Published:** 2018-05-19

**Authors:** Carly N. Cook, Carla M. Sgrò

**Affiliations:** ^1^ School of Biological Sciences Monash University Clayton VIC Australia

**Keywords:** adaptation, conservation management, gene flow, genetic diversity, inbreeding depression, outbreeding depression

## Abstract

Despite wide acceptance that conservation could benefit from greater attention to principles and processes from evolutionary biology, little attention has been given to quantifying the degree to which relevant evolutionary concepts are being integrated into management practices. There has also been increasing discussion of the potential reasons for a lack of evolutionarily enlightened management, but no attempts to understand the challenges from the perspective of those making management decisions. In this study, we asked conservation managers and scientists for their views on the importance of a range of key evolutionary concepts, the degree to which these concepts are being integrated into management, and what would need to change to support better integration into management practices. We found that while managers recognize the importance of a wide range of evolutionary concepts for conservation outcomes, they acknowledge these concepts are rarely incorporated into management. Managers and scientists were in strong agreement about the range of barriers that need to be overcome, with a lack of knowledge reported as the most important barrier to better integration of evolutionary biology into conservation decision‐making. Although managers tended to be more focused on the need for more training in evolutionary biology, scientists reported greater engagement between managers and evolutionary biologists as most important to achieve the necessary change. Nevertheless, the challenges appear to be multifaceted, and several are outside the control of managers, suggesting solutions will need to be multidimensional.

## 1 INTRODUCTION

There is a growing consensus among evolutionary biologists that better conservation outcomes could be achieved by considering evolutionary principles and process in the design of appropriate management actions (Carroll et al., 2014; Mace & Purvis, 2008; Sgrò, Lowe, & Hoffmann, 2011; Smith, Kinnison, Strauss, Fuller, & Carroll, 2014). Such an approach has been termed evolutionarily enlightened management (Carroll et al., 2014). Recent reviews have suggested a range of conservation management issues that would benefit from more evolutionarily enlightened management, including threatened species management, restoration projects and invasive species management (Frankham, 2010; Smith et al., 2014; Weeks et al., 2011). Recommendations for improved practice often centre on the active management of genetic diversity to maintain resilient populations (Table 1; Hoffmann & Sgrò, 2011; Sgrò et al., 2011; Weeks et al., 2011). While the goal of conserving genetic diversity is widely recognized within international conservation policies, such as the Convention on Biological Diversity, little, if any, specific direction is provided about how management practices need to change (Cook & Sgrò, 2017). Conservation policies within individual countries appear to do little more than mention genetic diversity, while more specific evolutionary concepts (evolutionary process and principles), such as inbreeding and outbreeding depression or gene flow, are almost entirely absent (Cook & Sgrò, 2017).

**Table 1:** Areas of environmental management in which evolutionary principles and processes could be used to inform management actions

Evolutionary principle or process	Threatened species management	Restoration and revegetation	Invasive species management	Protected area design	Wildlife disease management
Genetic diversity					
Genetic diversity provides the variation through which adaptation and evolution can occur. Genetic diversity increases effective population size, which is critical to the viability of populations. Small, fragmented populations are more vulnerable to the loss of genetic diversity	Threatened species tend to have small, fragmented populations, with low effective population sizes and vulnerability to loss of genetic diversity through genetic drift and inbreeding depression (Ellstrand & Elam, 1993). Genetic diversity must be actively managed within threatened populations, potentially by facilitating gene flow via maintaining connectivity between populations or translocating individuals (Weeks et al., 2011). Selecting individuals for captive breeding should focus on maximizing genetic diversity	To ensure the long‐term viability of restoration efforts, seed should be sourced from multiple populations (Broadhurst et al., 2008), preferably spanning an environmental gradient, to maximize genetic diversity and adaptive potential in the face of environmental change (Sgrò et al., 2011)	When managing invasive species, the goal is to minimize genetic diversity. Biosecurity measures can be used to avoid multiple introductions that increase the genetic diversity of invasive populations (Simberloff, 2009). Reducing population size will help to reduce genetic diversity. Wherever possible, manage the risk of hybridization between closely related native and exotic species that can increase genetic diversity and adaptive potential (Schierenbeck & Ellstrand, 2009)	Ensure protected areas can support sufficiently large populations to maintain genetic diversity (Mace & Purvis, 2008). Adequately protecting species may require protecting multiple populations along an environmental gradient to maximize the genetic diversity among populations (Lankau et al., 2011). Focusing on protecting areas that preserve evolutionary processes and have served as evolutionary refugia may help conserve genetic differences (Smith, Bruford, & Wayne, 1993)	Disease can reduce the size of affected populations, which will reduce genetic diversity. Higher levels of genetic diversity in affected populations may reduce the impact of novel diseases (Vander Wal, Garant, Calme et al., 2014). Management of disease should consider efforts to restrict the genetic diversity of pathogen populations
Adaptation					
Adaptation is the process by which selection acts on standing genetic diversity to enable species to respond to environmental change	Threats that drive species to become threatened are acting as selection pressures that can lead to changes, some of which may be maladaptive, for example, changes in flowering time in response to warming climates that disrupt mutualisms with pollinators (Merila & Hendry, 2014). Managing threats can reduce the impacts they have on the fitness of threatened species (Hendry et al., 2011). Captive breeding programmes must be managed to reduce the risk that individuals become adapted to captive rather than natural conditions (Williams & Hoffman, 2009)	Current conditions may not represent future conditions, so ensuring revegetated areas have a mixture of locally adapted seed, and seed from populations adapted to conditions similar to those expected to arise under climate change scenarios could improve the adaptive potential and therefore resilience of revegetated areas (Sgrò et al., 2011)	Management activities function as a selection pressure on invasive populations, which could lead to adaptations that reduce management effectiveness (Neve et al., 2014). This is particularly a problem for the use of toxins (e.g., herbicides, pesticides). Rotating or stacking toxins can help to avoid strong directional selection that leads to rapid adaptation (Beckie & Reboud, 2009). Adaptation can have costs that should be considered when designing control programmes (Lagator, Vogwill, Colegrave, & Neve, 2013)	The same considerations as for genetic diversity	Ensure treatment of pathogens is performed in a way to reduce adaptations that reduce the effectiveness of control measures (Smith et al., 2014). Using management activities to select for resistant individuals can help minimize the impact of disease on wildlife (Vander Wal, Garant, & Pelletier, 2014)
Evolution					
Evolution is the result of changes in allele frequency within a population over generations. It is important to understand which populations will be able to adapt and which risk extinction. Evolutionary history is the product of past selection pressures, and understanding this offers insights into constraints on adaptation and how it can be used to control undesirable species	Maintaining or increasing genetic diversity, and therefore adaptive potential, is critical to rapid or contemporary evolution, which can enable species to respond to rapidly changing conditions under a range of anthropogenic pressures (Hendry, Farrugia, & Kinnison, 2008)	The same consideration as for adaptation	Management actions should consider how to limit the adaptive capacity of invasive populations and to reduce directional selection that leads to adaptations that resist control efforts (Hendry et al., 2017). Biological control programmes can utilize evolutionary history by employing pests that have evolved to limit exotic populations within their native range (Van Klinken & Edwards, 2002). Programmes can also support native insects to evolve increased ability to attack introduced plants (Carroll, 2011)	The same consideration as for genetic diversity	Pathogens can evolve resistance to treatments. Costs of adaptation can be used to manipulate evolutionary trajectories through the design of treatments that slow the evolution of resistance (Levin, Perrot, & Walker, 2000)
Gene flow					
Gene flow through the movement of individuals between populations can introduce new alleles that increase the genetic diversity of populations. Gene flow can benefit populations by increasing effective population size, counteracting genetic drift, reducing inbreeding depression and introducing favourable alleles. Gene flow can also be negative if it works to disrupt local adaptation or facilitates adaptation of pest species	The small, often fragmented nature of threatened populations means that gene flow is important to maintain or restore genetic diversity (Weeks et al., 2011). Where connectivity has been disrupted, managed gene flow through translations can act to restore genetic diversity and reverse the impacts of inbreeding depression (e.g., genetic rescue; Weeks et al., 2017)	Sourcing seed from populations that occur along an environmental gradient can maximize the adaptive capacity and resilience of restoration effects (Prober et al., 2016). Maximizing connectivity along an environmental gradient can enable the movement of adaptive alleles (Jordan, Hoffmann, Dillon, & Prober, 2017)	Restrict gene flow between populations to limit genetic diversity and adaptive potential (Hendry et al., 2011). It is important to assess the risk that genetically modified organisms transfer herbicide resistance to invasive species (Andow & Zwahlen, 2006). Use gene flow to disrupt the evolution of resistance by increasing gene flow from susceptible populations (Tabashnik et al., 2013)	Ensure protected areas are well connected to facilitate gene flow among populations (Ridley & Alexander, 2016). Situating protected areas to allow gene flow across environmental gradients can help facilitate adaptation to changing environments (Lankau et al., 2011)	Gene flow can serve to increase the adaptive capacity of pathogens, but can also be used to disrupt the evolution of resistance by increasing gene flow from susceptible populations (Alphey, Coleman, Donnelly, & Alphey, 2007)
Inbreeding depression					
Inbreeding depression, the decreased fitness of offspring due to increased levels of homozygosity leading to the expression of deleterious recessive alleles, leads to loss of genetic diversity and demographic suppression	Small, isolated populations are prone to inbreeding depression, which can increase extinction risk (Hedrick & Kalinowski, 2000). The risk of inbreeding depression must be managed in captive breeding programmes, which can have low effective population sizes. Facilitating gene flow from populations with higher levels of genetic diversity can reverse the negative impacts of inbreeding depression (Weeks et al., 2017)	Where local populations are small and fragmented, sourcing seed locally can lead to low levels of genetic diversity, and therefore a higher risk of inbreeding depression (Prober et al., 2016)	Keeping populations small and isolated will increase the chance of inbreeding depression. This can be achieved through population reduction, limiting gene flow and prioritizing management early in the invasion process before species become widespread (Simberloff, 2009)	Protected areas should be large enough and/or sufficiently connected to avoid the risk of inbreeding depression associated with small, fragmented populations	Impact of novel diseases can reduce wildlife populations to the point they are vulnerable to inbreeding depression
Outbreeding depression					
Outbreeding between individuals from genetically distinct populations can lead to reduced fitness through genetic incompatibility and/or maladaptation	The risk of outbreeding depression should be considered when planning translocations, using available risk assessment frameworks (Frankham et al., 2011)	The risk of outbreeding depression should be considered when selecting seed for restoration efforts, using available risk assessment frameworks (Frankham et al., 2011)	Not currently being discussed in the literature	Not currently being discussed in the literature	Not currently being discussed in the literature
Mating system					
Different mating systems are associated with different levels of genetic diversity and impact the relationship between census and effective population size	Different mating systems are associated with different levels of risk of inbreeding and outbreeding depression (Weeks et al., 2011). The type of mating system should inform estimates of viable population size (Lankau et al., 2011)	Mating systems should be considered when selecting how many populations to source seed from (e.g., genetic diversity is lower in populations of selfing or asexual reproducers; Frankham, 2010). Revegetation can pose a genetic risk of hybridization where related species have compatible mating systems (Byrne et al., 2011)	Mating systems influence genetic diversity (e.g., lowest in asexual reproducing species) and therefore the adaptive capacity of invasive species (Frankham, 2010)	Not currently being discussed in the literature	Not currently being discussed in the literature
Life history strategy					
Life history traits, such as generation time, play a critical role in how quickly species can adapt to environmental change. Life history strategy also impacts the relationship between census and effective population size (e.g., when individuals reach sexual maturity)	Life history traits influence the ability of species to adapt to changing environmental conditions (Hendry et al., 2011), influencing their vulnerability to extinction (Gallagher, Hammerschlag, Cooke, Costa, & Irschick, 2015). Life history information can inform estimates of viable population size (Lankau et al., 2011), appropriate rates and timing of gene flow (i.e., translocation rate; Weeks et al., 2011), etc	It can take a long time to realize the impact of low genetic diversity in revegetation efforts for species with long generation times (e.g., tree species; Prober et al., 2016)	Biosecurity measures should target species that can produce large numbers of propagules because they are most likely to become invasive (Simberloff, 2009)	Protected areas can be used as refuges for exploited species to reduce the impacts of life history evolution driven by selective harvesting (Baskett, Levin, Gaines, & Dushoff, 2005)	Understanding life history strategy can inform the timing of treatments because those that act after first reproduction will slow the evolution of resistance in pathogens (Hendry et al., 2011)

The evidence of poor integration of evolutionary concepts into conservation policy documents (e.g., Cook & Sgrò, 2017; Lankau, Jorgensen, Harris, & Sih, 2011; Pierson et al., 2015) supports the widespread criticism from evolutionary biologists that conservation managers (policymakers and on‐ground managers) are not changing their management practices in response to the available science. A key concern raised by evolutionary biologists is the general lack of consideration given by managers to how their management practices can act as a selection pressure on wild populations (Hendry et al., 2011; Smith et al., 2014). This is particularly relevant for invasive species management, where the evidence from agricultural science can inform risk assessments for management practice that might promote the evolution of resistance to the toxins used to control pest species (Neve, Busi, Renton, & Vila‐Aiub, 2014; Tabashnik, Brevault, & Carriere, 2013). Despite continued commentary about the long‐term implications of management actions that do not consider evolution (Carroll et al., 2014; Hoffmann & Sgrò, 2011; Smith et al., 2014), the reasons for this gap remain largely speculative. Some of the many reasons proposed by evolutionary biologists to explain the widespread failure to integrate evolution into management practices include poor training of managers in evolutionary biology (e.g., Frankham, 2010) and a lack of support for conservation managers to enable them to change their management practices (e.g., Hoban et al., 2013; Smith et al., 2014).

Anecdotal reports describing misconceptions among managers about evolutionary theory, which might act as impediments to evolutionarily enlightened management, have been blamed on a lack of specific training for conservation managers. For example, some authors have reported that managers often view species as fixed entities that do not change (Ashley et al., 2003), or believe that evolution happens too slowly to be relevant to management practices (Kinnison, Hendry, & Stockwell, 2007; Smith et al., 2014). It has also been suggested that managers have risk adverse attitudes to changing their management practices, viewing the manipulation of evolutionary forces as potentially disrupting the integrity of natural processes (Hendry et al., 2011; Smith et al., 2014). Concerns have also been raised that the actions of many managers remain guided by largely outdated or misinterpreted ideas, for example, anxiety about the risk of outbreeding depression when mixing populations, despite growing evidence that the risks have been overstated (Frankham, 2015; Frankham et al., 2011). Similarly, the focus on the “local is best” paradigm, which places precedence on collecting seed from populations considered to be adapted to local conditions, may limit the genetic diversity of revegetated populations, compromising their long‐term viability and success (Broadhurst et al., 2008; Byrne, Stone, & Millar, 2011). However, despite concerns that conservation managers misunderstand evolutionary concepts, the evidence for this belief remains anecdotal; managers’ knowledge of evolutionary biology has not been quantified.

Some authors have identified a lack of appropriate decision‐support tools as a barrier to better integration of evolutionary biology into conservation management (Frankham, 2010; Hoban et al., 2013). Specifically, changes to management practice require the development of tools that enable managers to identify the practical changes needed for evolutionarily enlightened management. However, criticisms have been made that evolutionary theory continues to be ignored within existing decision tools, such as population viability analysis (Ashley et al., 2003; Frankham, 2010) and species distribution models (Urban et al., 2016). In other cases, there is a failure to translate theory into practical management recommendations that managers could implement (Cook & Sgrò, 2017). For example, recommendations that managers should monitor genetic diversity are increasingly the norm (Cook & Sgrò, 2017), yet practical guidance about how this should be done and how to distinguish between neutral and adaptive diversity is largely lacking (Hoffmann et al., 2015). It has also been suggested that the slow integration of evolutionary biology into conservation practice may stem from a general failure by evolutionary biologists to engage with conservation managers (Hendry et al., 2010; Hoban et al., 2013; Mace & Purvis, 2008).

The increasing attention in the literature given to the need for evolutionarily enlightened management, and the potential reasons for this slow uptake have helped raise awareness of this problem in conservation biology (Hendry, Gotanda, & Svensson, 2017). However, moving beyond the current speculation requires a clear understanding of the scale of the problem, quantifying the degree to which different evolutionary concepts are being integrated into management practice. Likewise, these data need to be paired with evidence for the causes of poor uptake in order to identify the most efficient ways to achieve change. This evidence needs to come from the conservation management community, to gain a true appreciation of the challenges they face in making changes to management practices. Because this evidence is currently lacking, our objective was to fill this important gap by surveying conservation managers, along with conservation scientists involved in conducting management‐relevant science, about their views on (i) the importance of a range of key evolutionary concepts to conservation management, (ii) the current level of integration of these concepts into management decisions and (iii) the barriers and opportunities they see to achieving the necessary change.

## 2 MATERIALS AND METHODS

To explore the degree to which evolutionary biology has been integrated into conservation management, we developed an anonymous, online questionnaire (Appendix 1) focused on a set of key evolutionary concepts (Table 2). These concepts were selected to represent evolutionary processes and principles from evolutionary theory and population genetics that are considered to be highly relevant to conservation management decisions. The selection of the concepts was informed by the frequency of their mention in the literature and in consultation with experts in applied evolutionary biology (withheld for review).

**Table 2:** Key concepts relevant to integrating evolutionary theory into conservation practice

Concept	Definition
General concepts	
Genetic diversity	Genetic differences between individuals of the same species
Adaptation	The condition where the phenotype of individuals is well suited to the environmental conditions, such that the individuals have higher reproductive fitness.
Evolution	The process by which populations or species change over successive generations.
Specific concepts	
Gene flow	Movement of alleles between populations through mating between individuals from different populations
Inbreeding depression	Mating between closely related individuals that leads to a loss of genetic diversity and corresponding reduction in reproductive fitness
Outbreeding depression	Mating between genetically distinct individuals that introduces new alleles that disrupt local adaptation and lead to reduced reproductive fitness
Mating system	The way in which a population is structured in relation to sexual behaviour
Life history strategy	The way in which individuals invest in growth, reproduction and survivorship

We targeted two groups of respondents:

1. conservation managers in policy or on‐ground management roles; and
2. applied ecologists whose research programmes focus on management‐relevant science.

The conservation managers were protected area or natural resource managers drawn from across all jurisdictions in Australia (five states, two territories and the federal government). Senior managers within protected area management agencies (*n* = 8) and national resource management organizations (*n* = 56) were contacted and asked to distribute the link to an online questionnaire to all relevant staff members, including those in policy and on‐ground management roles.

We also contacted scientists whose research addresses conservation management problems. These individuals were identified by searching staff profiles from universities and government research institutes across Australia (*n* = 23). Relevant individuals were contacted via email (*n* = 78) and invited to participate in the study. They were also asked to distribute the survey to relevant members of their research groups, following a snowball sampling approach (Patton, 2002).

### 2.1 Questionnaire development

We asked respondents to determine how important each of the key concepts in Table 2 was for conservation management, and to what degree they believed each concept was being integrated into current management practices. Respondents were asked to score their responses according to a 5‐point Likert scale (Table 3), with an option to indicate if they were unsure about any of the concepts. Respondents were not provided with definitions of the concepts, in order to avoid any temptation to speculate about the importance or integration of concepts they were not familiar with. However, this would not preclude respondents from looking up the meaning of concepts they were unsure about. It may also mean that respondents had varying interpretation of the concepts, which may have influenced their responses.

**Table 3:** The 5‐point Likert scale used by respondents to score their responses to the questionnaire

Importance	Integration	Coding
Not at all important	Never	1
Somewhat important	Rarely	2
Neither important nor unimportant	Sometimes	3
Important	Often	4
Very important	All the time	5
Unsure	Unsure	Missing value

We also asked respondents to describe their perception of the most important barriers that inhibit integration of evolutionary theory into conservation management, and what they believed would need to change within their organizations for evolutionary theory to be adopted more broadly. Respondents were asked to provide a range of demographic information, including their role, age, years of experience, gender, level of education and exposure to evolutionary theory during their formal education (Appendix 1). Scientists were also asked whether they spend time engaging with managers about effective management practices.

Before distributing the questionnaire, the tool was piloted with seven individuals who confirmed the face validity (i.e., whether the meaning of the questions is clear to respondents and results of the survey will provide a meaningful outcome; Wainer & Braun, 1988) of the survey tool.

### 2.2 Data analyses

We coded responses (Table 3) and used Kruskal–Wallis nonparametric tests to determine whether respondents differed in how they ranked the importance of each concept for conservation management, and in the degree to which they were integrated into management decisions. To determine whether there were differences in the level of importance or in the level of integration assigned to each concept by managers and scientists, we used Mann–Whitney U nonparametric tests. Kruskal–Wallis nonparametric tests were used to compare respondents’ views on the level of importance and level of integration of the concepts with whether they had received formal training in the relevant concepts (i.e., subjects in evolutionary biology or genetics).

The questions relating to barriers and opportunities for better integration of evolutionary theory were grouped according to common themes in their responses (i.e., open‐coded following an inductive category development methodology; Patton, 2002). All responses were coded independently by two researchers to ensure consistent interpretation. All quantitative analyses were performed in SPSS version 23.

## 3 RESULTS

We received 150 responses to the questionnaire (107 managers; 43 scientists) drawn from across Australia. Managers were mostly in on‐ground management (*n* = 58) or policy or strategy roles (*n *= 39) (10 did not indicate their role). These respondents were mostly males (57%) and ranged in experience from 6 months to 29 years (μ = 8.7). The scientists who responded were in research only (*n* = 18) or teaching and research roles (*n* = 21), and just over half of scientists (56%) indicated that they engaged directly with managers. Most scientists were female (56%), and their experience ranged from 1 to 43 years (μ = 9.3).

The level of education of managers varied from high school graduates to postgraduate degrees (*n* = 6 declined to answer), while scientists had a bachelor's degree or higher (*n* = 4 declined to answer; Figure S1). Most respondents in each group reported having taken at least some subjects relevant to evolutionary theory, although 34% of managers had not (Figure S1). Ten managers and four scientists declined to answer.

### 3.1 The importance of evolutionary concepts

**Figure 1:**
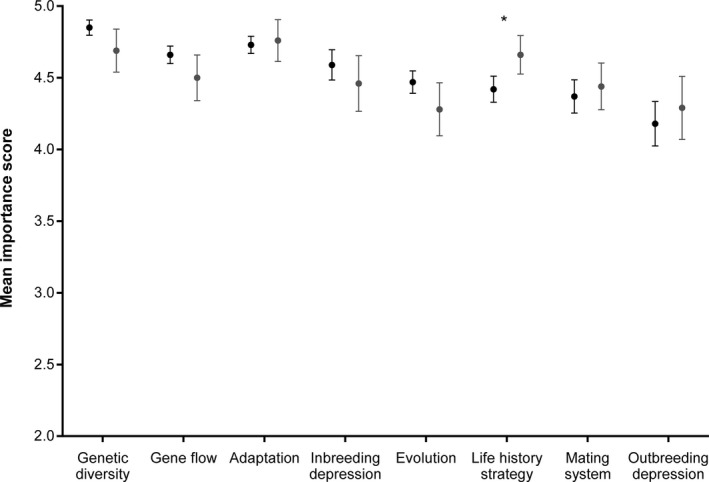
The mean (±*SE*) importance score for different evolutionary concepts as reported by managers (black circles) and scientists (grey circles). Asterisk indicates significant difference between groups

There were significant differences in the level of importance assigned by respondents to the different evolutionary concepts (*H*_7_ = 46.70; *p* < .001). While there was variation in which concepts managers and scientists considered most important to conservation management, this difference was only significant for the importance of life history strategy (*U*_2_ = 588.0; *z* = −2.02; *p* = .044; Figure 1; Table S1). Among managers, there was no difference in the level of importance ascribed to the evolutionary concepts by those in strategic roles and those in on‐ground roles (*U*_2_ = 24676.00; *z* = −0.43; *p* = .668; Table S2).

**Figure 2:**
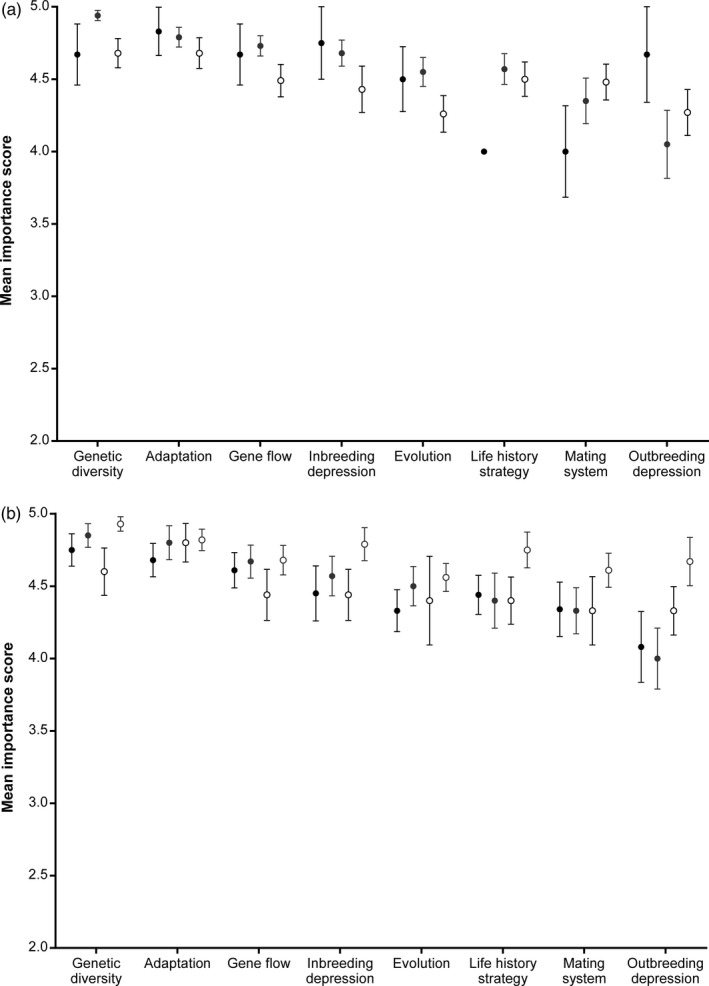
The mean (±*SE*) importance score for each of the evolutionary concepts based on (a) the level of education of respondents (black circles = certificate/diploma; grey circles = bachelor; open black circles = postgraduate) and (b) the level of exposure to evolutionary biology during their training (black circles = evolution and genetics; grey circles = evolution only; open black circles = genetics only; open grey circles = none)

Respondents with different levels of education had significantly different views on how important the different concepts are to conservation management (*H*_3_ = 9.32; *p* = .025; Figure 2a), with greater importance ascribed by those with a diploma or bachelor's degree. Prior exposure to evolutionary theory during their education influenced how important respondents considered evolutionary concepts to be (*H*_3_ = 10.71; *p* = .013; Figure 2b), with those who indicated they had only taken subjects relating to genetics being less likely to consider the concepts important. Further evidence that lower importance scores may relate to the level of understanding of these concepts comes from the correspondence between more respondents failing to score the importance of a concept (i.e., selecting unsure) and a lower importance score ascribed to the concept by other participants (Table S3).

### 3.2 Integration of evolutionary concepts

**Figure 3:**
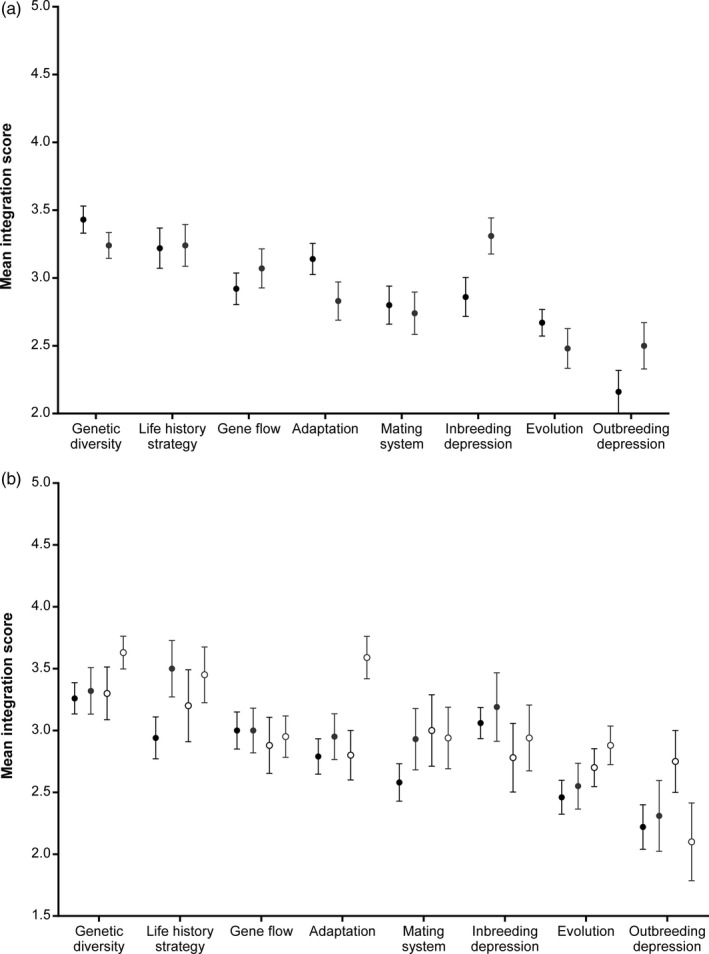
The mean (±*SE*) integration score for each of the evolutionary concepts as reported by (a) managers (black circles) and scientists (grey circles), and (b) the level of exposure to evolutionary biology during their training (black circles = evolution and genetics; grey circles = evolution only; open black circles = genetics only; open grey circles = none)

There was a significant difference in the degree to which respondents considered the different evolutionary concepts were being integrated in conservation management (*H*_7_ = 77.43; *p* < .001). While there was variation in the views of managers and scientists about the degree to which different concepts were being integrated into conservation management, overall these differences were not significant (U_2_ = 49409.0; *z* = −0.38; *p* = .705; Figure 3a; Table S4). Within managers, those in policy or strategy roles and those in on‐ground roles had similar views of how well the evolutionary concepts were implemented within conservation management (U_2_ = 23416.0; *z* = −1.56; *p* = .118; Table S5), while scientists who indicated they engaged directly with managers tended to show closer alignment to the views of managers (Figure S2).

The level of education of respondents did not influence the degree to which they believed evolutionary concepts were being integrated (*H*_3_ = 1.70; *p* = .638). However, those who had not taken subjects specifically related to evolutionary biology during their degrees considered the concepts to be better integrated into conservation management (*H*_3_ = 16.38; *p* = .001; Figure 3b).

### 3.3 Barriers to and opportunities for greater integration of evolutionary theory

**Figure 4:**
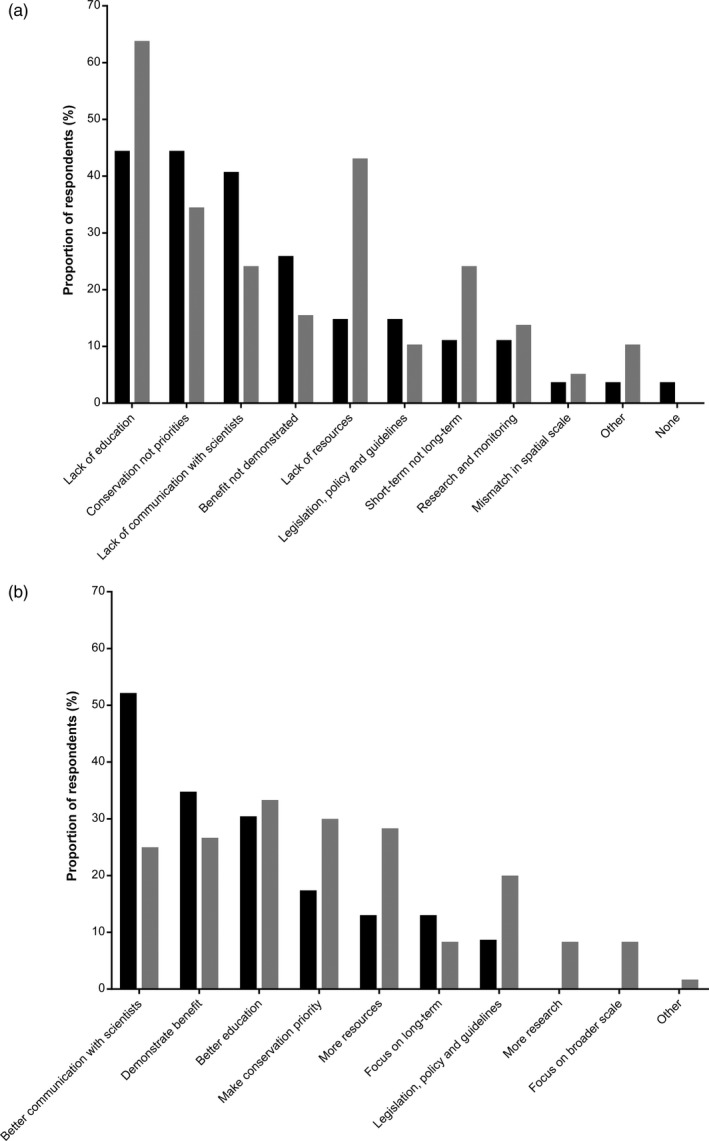
The (a) barriers to and (b) opportunities for greater integration of evolutionary theory into conservation management as reported by scientists (black bars) and managers (grey bars)

The most commonly reported barrier to greater integration of evolutionary theory by managers and scientists was a lack of understanding of the concepts (Figure 4a). Respondents from both groups also commonly suggested that conservation management is not being prioritized within management agencies, with an increasing emphasis being given to visitor management (Figure 4a; Table 3). Managers showed greater concern with a lack of resources to implement the necessary management actions, while scientists ranked a lack of communication between managers and researchers more highly (Figure 4a). Scientists were more concerned that a failure to demonstrate clear benefits of changed management practices was a barrier, while managers were concerned that a focus on short‐term outcomes of management prevented greater integration of evolutionary theory (Figure 4; Table 4).

**Table 4:** Description of the barrier to better integration of evolutionary theory reported by managers and scientists

Category Code	Description
Barriers to better integration	
Lack of education	A lack of training and basic understanding of the relevant concepts, their importance and/or how they should be applied to management
Conservation not prioritized	A lack of support for conservation from governments and the broader community, and a shift in emphasis to towards visitor management rather than conservation management
Lack of resources	Declines in the resources for conservation management mean funds are inadequate for on‐ground management. There are competing priorities for resources, and funding is uncertain and short‐term. Too few managers.
Lack of communication with scientists	A lack of engagement by scientists means there is poor translation of primary research into management programmes
Short‐term not long‐term	The focus of management is on dealing with immediate problems, not long‐term outcomes. There is poor understanding of how concepts can be applied to short management time frames. Evolution and management occur over different time horizons.
Benefit not demonstrated	There is little evidence for the application of relevant concepts to conservation management. There are no case studies that show chances are beneficial.
Research and monitoring	Not enough research or funding for necessary research. Research is generally academic and not focused on conservation management. Unclear what to monitor.
Legislation, policy and guidelines	Legislation is interpreted too narrowly and provides impediments (e.g., managing across borders) to including evolutionary processes. There is no mandate within policy to change management practices. Managers do not know how to integrate these ideas into their practices.
Other	Conflict between evolutionary theory and religious beliefs. Need to engage other landholders and managers for integrated management.
Mismatch in spatial scale	Management occurs at small scales (e.g., small, isolated areas), but evolutionary process often need to be managed at a landscape scale.
None	There is currently nothing preventing greater integration.

The opportunities identified for greater integration of evolutionary theory into conservation management were directly related to the key barriers, essentially being potential solutions (Figure 4b). Managers and scientists differed somewhat in which solutions they saw as highest priority to improve the uptake of evolutionary theory. Scientists were more focused on greater engagement with managers and the need to demonstrate the benefits of changed management practices in improving conservation outcomes (Figure 4b). Managers tended to be more focused on the need for more training in evolutionary biology, a greater emphasis on conservation management within their agencies and additional funding to facilitate the required changes (Figure 4b).

## 4 DISCUSSION

Despite increasing discussion in the literature about evolutionary biology not being integrated into conservation management (Carroll et al., 2014; Frankham, 2010; Mace & Purvis, 2008; Smith et al., 2014), this study is the first to address the challenge from the perspective of the conservation managers and the scientists who engage with them. We reveal that most managers strongly support the view that evolutionary biology is important for conservation management (Figure 1), but demonstrate there is a long way to go to achieve routine integration of these concepts into management practices (Figure 3). Our data also clearly show that managers face a range of practical constraints in trying to achieve the required changes (Figure 4). By identifying these barriers and opportunities, we provide important insights into how the scientific community can better support managers, but also the changes that must occur within management organizations, and society more generally, to achieve real change.

### 4.1 Importance of evolutionary concepts and their integration into conservation management

Managers consider evolutionary biology to be highly relevant to their management practices (Figure 1), suggesting that the concerns raised by evolutionary biologists have been heard. In particular, the messages about the importance of general evolutionary concepts, such as genetic diversity (Hoban et al., 2013; Santamaria & Mendez, 2012; Sgrò et al., 2011; Weeks et al., 2011) and adaptation (Hoffmann & Sgrò, 2011), appear to have reached managers. These general concepts have also received more attention than other concepts within the evolutionary applications literature (Cook & Sgrò, 2017). Interestingly, evolution as a concept was not reported to be as important as adaptation and genetic diversity, potentially supporting concerns that rapid evolution is not being perceived as relevant to management (Ashley et al., 2003; Kinnison et al., 2007; Smith et al., 2014). This pattern also seems to suggest a poor understanding of the links between genetic diversity, adaptive capacity and evolution, which has also been observed in conservation policy (Cook & Sgrò, 2017).

While some evolutionary concepts were considered more important to conservation management than others, we found evidence that these differences may have been driven by poorer understanding of the relevance of some concepts. Managers who had a greater exposure to evolutionary biology in their training gave greater emphasis to the importance of these concepts (Figure 2b), supporting calls for the value of increased training for managers (Frankham, 2010). We also saw qualitative evidence for a lack of understanding of some concepts because managers were more likely to abstain from rating the importance for concepts that were given lower importance across the rest of the sample (Table S3). This may suggest a reluctance by respondents to rate concepts with which they were less familiar. However, without asking respondents to define each of the concepts, it is not possible to truly judge their specific level of understanding, or the consistency of interpretations among respondents. Poor understanding of evolutionary biology has been repeatedly suggested as a barrier to evolutionarily enlightened management (Ashley et al., 2003; Frankham, 2010; Mace & Purvis, 2008). Our results suggest that increased training in evolutionary biology may increase the value managers place on these concepts, and possibly their potential to integrate them into management.

There have been concerns expressed in the literature that managers are reluctant to mix individuals from different populations (e.g., gene pool mixing; Weeks et al., 2011) due to concerns about maladaptation of subsequent generations as a result of outbreeding depression (Frankham et al., 2011). However, the lower importance ascribed to outbreeding depression and the poorer application of the concept to management do not support this being a major concern for managers (Figures 1 and 2). The finding that managers consider outbreeding depression to be the least important concept for management decisions may reflect a poor understanding of the concept. We found that more than half of managers in the sample chose not to provide an importance score for outbreeding depression (Table S3), suggesting most managers were unfamiliar with this concept. The poor integration of outbreeding depression into management decisions suggests managers are also not aware of the risk assessment frameworks available to guide decisions about when to mix populations (Frankham, 2015; Weeks et al., 2011). These decision‐support tools also appear to have little penetration into conservation policy (Cook & Sgrò, 2017), making recent work that demonstrates the benefits of gene pool mixing (Frankham, 2016; Weeks et al., 2017) all the more important.

Both managers and scientists consistently considered the integration of evolutionary concepts to be out of step with their importance to management, with concepts rarely being integrated into management decisions (Figure 2). This supports the view that the level of integration of evolutionary concepts is generally quite poor (Hoban et al., 2013; Mace & Purvis, 2008; Smith et al., 2014). Both managers and scientists were in agreement that the greatest discrepancy between the importance and implementation of concepts occurs for outbreeding depression and evolution (Figures 1 and 2). Management agencies were considered to be doing better at integrating genetic diversity and life history strategies into management decisions (Figure 2), and there is certainly increasing guidance in the literature for how managers can take account of these concepts within revegetation (Breed, Stead, Ottewell, Gardner, & Lowe, 2013; Byrne et al., 2011; Sgrò et al., 2011) and threatened species management (Frankham et al., 2011; Weeks et al., 2011), which may be filtering through to managers. However, further work is needed to understand exactly how managers are applying these concepts to determine whether more effective long‐term management outcomes are likely to be achieved.

### 4.2 Changes required to facilitate greater integration of evolutionary theory into conservation management

While the message that evolutionary theory can benefit effective management appears to have been received by conservation managers, we found that both managers and scientists perceive a wide range of barriers that will need to be overcome to achieve greater integration (Figure 4a). Some of these barriers are within the control of scientists and managers to address, such as a lack of understanding of the relevant concepts (Ashley et al., 2003; Frankham, 2010) and poor engagement between scientists and managers (Hoban et al., 2013; Mace & Purvis, 2008). However, some barriers are beyond the control of both groups, such as insufficient resources to make changes to existing management practices (Figure 4a). This is not surprising, given the widely acknowledged shortfall in funding for conservation management (Waldron et al., 2013) is frequently cited as a barrier to implementing more effective management (Addison, Cook, & Bie, 2016; Leverington, Costa, Pavese, Lisle, & Hockings, 2010).

Other barriers to greater integration of evolutionary biology that are beyond the control of managers and scientists include a shift in the emphasis of management roles away from biodiversity towards visitor management (Figure 4). The concern that conservation management is being given lower priority by politicians (Addison, Flander, & Cook, 2017; Addison et al., 2016) and the general community (McCallum & Bury, 2013) seems to be translating to more prominence for tourism and economic objectives for natural areas, particularly within protected areas (Balmford et al., 2009; Eagles, 2002). Managers reported that the ability to achieve best practice in management is being impeded by this lack of support, an idea that scientists appear to corroborate (Figure 4a). This reduced emphasis on conservation management may also be reflected in some managers’ concerns that conservation management is overly focused on short‐term outcomes (Figure 4a), at the expense of the longer term benefits that can be achieved (Sgrò et al., 2011; Smith et al., 2014). While a focus on longer term outcomes is certainly important, this must be balanced with the understanding that evolution can occur at management‐relevant timescales (Kinnison et al., 2007), and negative outcomes can occur rapidly (Smith et al., 2014). Greater engagement between evolutionary biologists and conservation managers may need to focus on how to achieve a balance between managing immediate threats and achieving the best long‐term outcomes.

While we found broad agreement between managers and scientists about the barriers preventing greater integration of evolutionary theory in conservation, the two groups placed slightly different emphasis on the solutions needed to achieve change (Figure 4b). Managers focused on the need for more training to better understand evolutionary biology, while scientists emphasized the need to improve the science–policy interface to better support managers to make good decisions (Figure 4b). These two solutions could be seen as two sides of the same coin and have certainly been suggested as a key plank in improving the integration of evolutionary theory (Frankham, 2010; Mace & Purvis, 2008), and scientific evidence more generally (Cook, Mascia, Schwartz, Possingham, & Fuller, 2013), into conservation decisions. Evolutionary biologists have arguably been less proactive than ecologists when it comes to engaging with conservation managers about improving management practices (Hendry et al., 2010; Hoban et al., 2013; Smith & Bernatchez, 2008). Therefore, by playing a greater role in the science–policy interface, evolutionary biologists could help to significantly improve management practices and outcomes.

Both groups also suggested that there needs to be greater emphasis on research that demonstrates how better conservation outcomes can be achieved by changing management practices (Figure 4b). Many of the scientific advances in evolutionary theory have come through research on model systems, which allow greater traction when testing the impact of evolutionary processes (Frankham, 2015). However, it may be hard for managers to translate this evidence into their management contexts, or even to see the relevance of these studies to management (Cook & Sgrò, 2017; Frankham, 2010; Hoban et al., 2013). The evolutionary biology community appear to be heeding calls to demonstrate how theory can be translated into more effective management practices (Frankham, 2010; Hoban et al., 2013; Lankau et al., 2011), with more experimental studies being undertaken that demonstrate the benefits of evolutionarily enlightened management within conservation contexts (e.g., Frankham, 2015; Hedrick & Fredrickson, 2010). The increasing prevalence of these studies and risk assessment frameworks should assist managers to better understand the benefits that can be achieved through evolutionarily enlightened management. The important literature from other branches of natural resource management (e.g., forestry, fisheries and agriculture) should also not be ignored, offering insights into how to manage herbicide resistance (e.g., Neve & Powles, 2005) and climate change adaptation (Aitken & Whitlock, 2013), for example. Drawing managers’ attention to the findings of these studies could help demonstrate the practical application of what could be perceived to be more abstract theory. Greater interaction between managers and evolutionary biologists would support greater knowledge transfer and potentially provide a platform for research demonstrating the outcomes of alternative management practices.

## 5 CONCLUSIONS

Improving the uptake of evolutionary biology within conservation management requires a detailed understanding of the current level of integration into management practice. However, this knowledge must be paired with the views of conservation managers on the value they place on evolutionary theory, their level of understanding of relevant concepts and what needs to change to facilitate greater uptake. We reveal that managers do generally understand the importance of evolutionary theory to conservation management, although managers hinted through their survey responses that there are some concepts (e.g., outbreeding depression) that they do not fully understand. Further investigation of the depth of understanding of core evolutionary concepts would help to better assess the extent to which limited understanding acts as a barrier to evolutionarily enlightened conservation. We also show that managers face many barriers to improving the currently poor level of integration of evolutionary concepts into their management practices. There are practical things that can be done to improve the adoption of evolutionary theory, such as increasing the exposure of managers to evolutionary theory during their training, and greater engagement with managers by evolutionary biologists. However, there are also issues beyond the control of managers, such as the level of public and political support for conservation management and the resources available to improve management practices, which require more systemic changes. Likewise, when making management decisions, managers must also consider scientific evidence relating to a range of other disciplines (e.g., ecology, hydrology, geology), in addition to influential social, political and economic factors, which may limit their ability to achieve evolutionarily enlightened management. Therefore, it is likely that a multidimensional approach is needed to achieve the necessary change.

## CONFLICT OF INTEREST

None Declared.

## DATA ARCHIVING STATEMENT

We have been granted an exemption from data archiving some or all of the data associated with this paper.

## APPENDIX 1

### 1.1

Questionnaire used to elicit managers’ and scientists’ perceptions on the integration of evolutionary concepts into conservation management

What type of organization do you work for?

1. Management‐focused
2. Research‐focused

*If management‐focused*:

Which of these categories best described the type of organization do you work for?

1. Park management
2. Catchment management
3. Other

How would you describe your role?

1. Predominantly on‐ground management
2. Predominantly policy development
3. Predominantly planning and strategic support
4. Other

*If research‐focused*:

How would you describe your primary role?

1. Research only
2. Teaching focused
3. Research and teaching
4. Other

Do you spend time engaging with management agencies about how to improve management practices?

1. Yes
2. No

*All respondents*:

How many years have you worked in your current role?

How old are you?

Gender

1. Male
2. Female

Which state or territory do you currently work for?

1. Commonwealth
2. Australian Capital Territory
3. New South Wales
4. Northern Territory
5. Queensland
6. South Australia
7. Tasmania
8. Victoria
9. Western Australia

What is your highest level of education?

1. High school
2. Diploma
3. Certificate
4. Bachelor
5. Higher degree
6. Other

Did you take any subjects relevant to genetics or evolution during your training?

1. None
2. Genetics
3. Evolution
4. Other

In your opinion, how important are these concepts to conservation management?

**Table:**

Concept	Not at all important	Somewhat unimportant	Neither important nor unimportant	Somewhat important	Very important	Unsure
Evolution						
Adaptation						
Genetic diversity						
Inbreeding depression						
Gene flow						
Outbreeding depression						
Mating system						
Life history						

To what degree do you believe these concepts are integrated into current management practices?

**Table:**

Concept	Never	Rarely	Sometimes	Most of the time	Always	Unsure
Evolution						
Adaptation						
Genetic diversity						
Inbreeding depression						
Gene flow						
Outbreeding depression						
Mating system						
Life history						

What do you believe are the greatest barriers to further integrating evolutionary theory into conservation management?

What do you believe would need to change for these ideas to be more broadly adopted?
