# Supplementary material for: Understanding managers’ and scientists’ perspectives on opportunities to achieve more evolutionarily enlightened management in conservation
Source: Evol Appl. 2018 May 19;11(8):1371–88. doi: 10.1111/eva.12631 (PMC6099810; doi:10.1111/eva.12631)
Supplement: Supplementary file 1 [file EVA-11-1371-s001.docx]

**Appendix B – Supplementary figures**

**(A)**

**
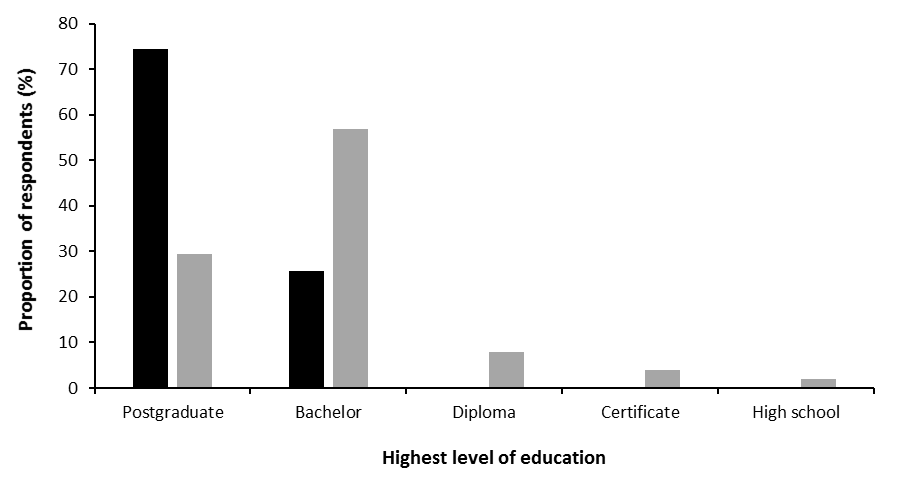
**

**(B)**

**
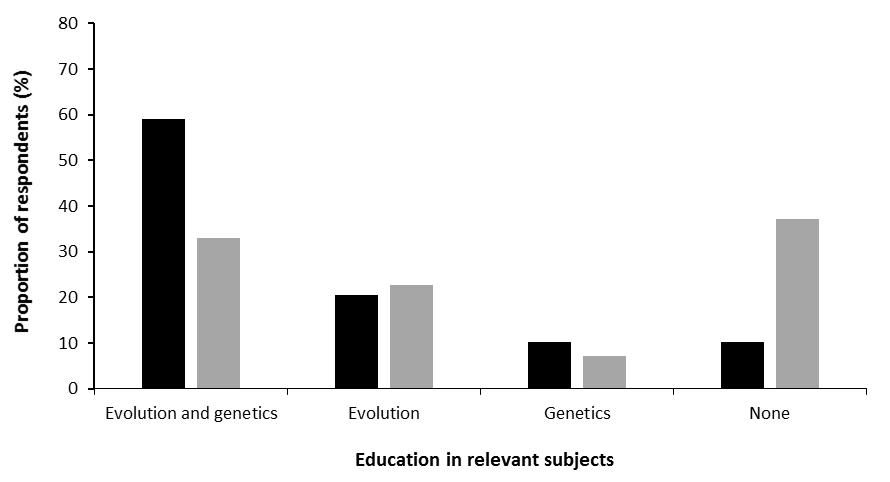
**

**Figure S1** – Level of: A) general education and B) specific training in relevant subjects of scientists (black bars) and managers (grey bars).

**
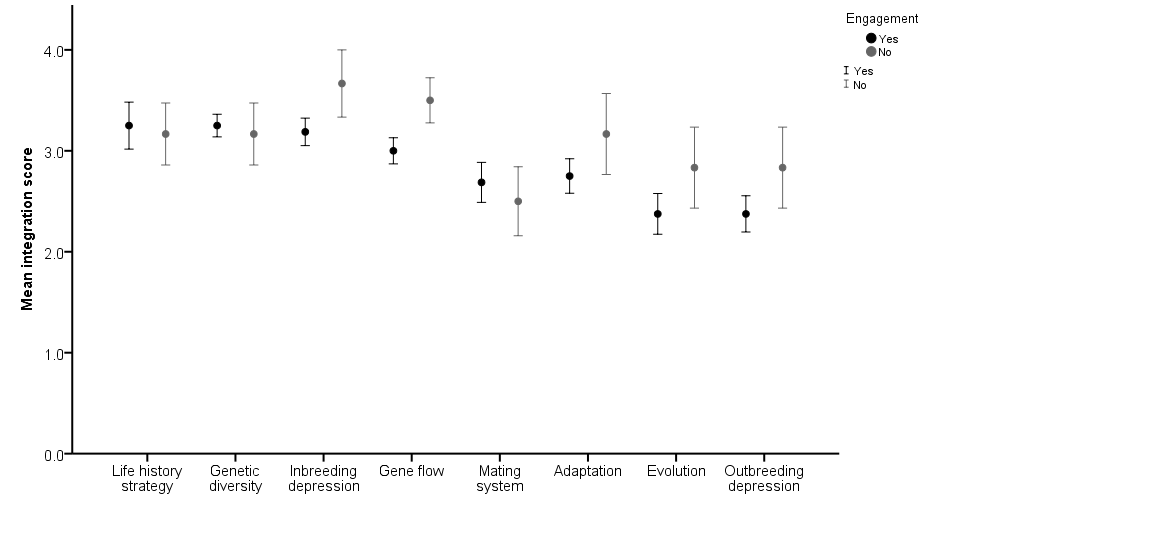
**

**Figure S2** – The mean (± SE) integration score for each of the evolutionary concepts as reported by scientists who do (black circles) and do not (grey circles) engage with managers

**Appendix C: Supplementary analyses**

**Table S1** – Results of Mann-Whitney U tests for differences in the views of managers and scientists on the importance of each evolutionary concept.

| **Level of importance of evolutionary concept** | **Mann-Whitney U** | **Z score** | **P-value** |
| --- | --- | --- | --- |
| Genetic diversity | 1012.50 | -0.37 | 0.709 |
| Adaptation | 930.50 | -1.06 | 0.288 |
| Evolution | 950.50 | -0.56 | 0.577 |
| Gene flow | 831.00 | -0.39 | 0.697 |
| Inbreeding depression | 588.00 | -0.37 | 0.709 |
| Outbreeding depression | 312.50 | -0.84 | 0.401 |
| Mating system | 606.50 | -0.67 | 0.503 |
| Life history strategy | 588.00 | -2.02 | 0.044* |

**Table S2** – Results of Mann-Whitney U tests for differences in the views of managers with a policy/strategy versus on-ground management roles on the importance of each evolutionary concept.

| **Level of importance of evolutionary concept** | **Mann-Whitney U** | **Z score** | **P-value** |
| --- | --- | --- | --- |
| Genetic diversity | 614.50 | -0.24 | 0.811 |
| Adaptation | 576.50 | -0.54 | 0.592 |
| Evolution | 583.00 | -0.12 | 0.902 |
| Gene flow | 456.50 | -0.10 | 0.922 |
| Inbreeding depression | 212.50 | -1.12 | 0.263 |
| Outbreeding depression | 127.00 | -0.32 | 0.749 |
| Mating system | 284.00 | -0.08 | 0.936 |
| Life history strategy | 332.00 | -0.11 | 0.910 |

**Table S3** – Correspondence between the frequency of non-response and mean importance scores for the different evolutionary concepts

| **Concept** | **Importance rank** | **Mean importance score** | **Frequency of non-response** |
| --- | --- | --- | --- |
| Genetic diversity | 1 | 4.80 | 47% |
| Adaptation | 2 | 4.74 | 33% |
| Gene flow | 3 | 4.61 | 40% |
| Inbreeding depression | 4 | 4.54 | 40% |
| Life history strategy | 5 | 4.50 | 45% |
| Evolution | 6 | 4.41 | 34% |
| Mating system | 7 | 4.39 | 49% |
| Outbreeding depression | 8 | 4.22 | 63% |

**Table S4** – Results of Mann-Whitney U tests for differences in the views of managers and scientists on the integration of each evolutionary concept.

| **Level of integration of evolutionary concept** | **Mann-Whitney U** | **Z score** | **P-value** |
| --- | --- | --- | --- |
| Genetic diversity | 824.00 | -1.52 | 0.129 |
| Adaptation | 799.00 | -1.68 | 0.093 |
| Evolution | 829.00 | -1.23 | 0.218 |
| Gene flow | 759.00 | -0.80 | 0.426 |
| Inbreeding depression | 506.50 | -1.78 | 0.075 |
| Outbreeding depression | 329.50 | -1.44 | 0.151 |
| Mating system | 638.50 | -0.41 | 0.681 |
| Life history strategy | 794.00 | -0.03 | 0.973 |

**Table S5** – Results of Mann-Whitney U tests for differences in the views of managers with a policy/strategy versus on-ground management roles on the integration of each evolutionary concept.

| **Level of integration of evolutionary concept** | **Mann-Whitney U** | **Z score** | **P-value** |
| --- | --- | --- | --- |
| Genetic diversity | 537.50 | -0.48 | 0.631 |
| Adaptation | 497.50 | -1.03 | 0.303 |
| Evolution | 503.50 | -0.57 | 0.569 |
| Gene flow | 410.00 | -0.36 | 0.719 |
| Inbreeding depression | 282.50 | -0.60 | 0.551 |
| Outbreeding depression | 140.50 | -0.97 | 0.333 |
| Mating system | 270.00 | -0.63 | 0.527 |
| Life history strategy | 318.50 | -1.03 | 0.305 |

**Appendix D – Opportunities to achieve greater integration of evolution into conservation management**

**Table S6** – Description of the opportunities for better integration of evolutionary theory reported by managers and scientists

| **Category Code** | **Description** |
| --- | --- |
| *Opportunities for greater integration* | |
| Better communication with scientists | Need to have evolutionary biologists involved in designing conservation plans and programs. Partner with research organisations or employ scientists. Run workshops with scientists. |
| Demonstrate benefit to management | Provide quality information on the value of changing management. |
| Better education | More training to increase awareness and the capacity of staff to integrate the ideas. Communicate successful case studies. |
| Make conservation a priority | Increased commitment to conservation over visitor management. Society needs to make conservation a priority |
| More resources | More staff and funding for management. Seek co-funding for conservation projects. |
| Increased focus on long-term management | Start considering outcomes over longer horizons than a few years. Develop longer term management plans. Move beyond reactionary management. |
| Improved legislation, policy and guidelines | Review legislation and policy frameworks. Seek greater support from government |
| Greater focus on broad scale management | Provide a greater emphasis on conservation management at large/landscape scales |
| More research | Invest more in scientific research. Need research that provides more concrete answers. |
| Other | Change the culture of organisations |
